# Emerging nanomaterials for dental treatments

**DOI:** 10.1042/ETLS20200195

**Published:** 2020-11-17

**Authors:** Zi Hong Mok, Gordon Proctor, Maya Thanou

**Affiliations:** 1 School of Cancer and Pharmaceutical Sciences, Faculty of Life Sciences and Medicine, King's College London, London, U.K.; 2 Faculty of Dentistry, Oral and Craniofacial Sciences, King's College London, London, U.K.

**Keywords:** endodontics, nanomaterials, orthodontics, periodontics, prosthodontics, remineralisation

## Abstract

The emergence of nanomaterials for dental treatments is encouraged by the nanotopography of the tooth structure, together with the promising benefits of nanomedicine. The use of nanoparticles in dentistry, also termed as ‘nanodentistry', has manifested in applications for remineralisation, antimicrobial activity, local anaesthesia, anti-inflammation, osteoconductivity and stem cell differentiation. Besides the applications on dental tissues, nanoparticles have been used to enhance the mechanical properties of dental composites, improving their bonding and anchorage and reducing friction. The small particle size allows for enhanced permeation into deeper lesions, and reduction in porosities of dental composites for higher mechanical strength. The large surface area to volume ratio allows for enhanced bioactivity such as bonding and integration, and more intense action towards microorganisms. Controlled release of encapsulated bioactive molecules such as drugs and growth factors enables them to be delivered more precisely, with site-targeted delivery for localised treatments. These properties have benefitted across multiple fields within dentistry, including periodontology and endodontics and reengineering of dental prosthetics and braces. This review summarises the current literature on the emerging field of nanomaterials for dental treatments.

## Introduction

Nanotechnology has allowed significant improvements in medicine and healthcare. Foreseeably, the development of nanomaterials has encouraged innovative applications in oral health. This is predominantly due firstly to the ability to mimic the nanostructure of the tooth surface and the nanosized organic components and secondly the inherent properties of nanomaterials [1,2].

Biomimetic nanotechnology emulates the nanostructure of the tooth enamel and surrounding proteins to achieve remineralisation [3]. Pioneering work has identified the average size of hydroxyapatite crystallites on enamel as 32.1 ± 3.5 nm long and 36.6 ± 1.7 nm wide [4]. The pore radius for sound enamel measures broadly between 1–30 nm [5]. Amorphous calcium phosphate is the precursor of hydroxyapatite, which arises from the nucleation of calcium and phosphate ions in saliva [6], aggregating to become spherical Posner's clusters (Ca_9_(PO_4_)_6_), reported to be 0.9 nm in size [7]. Amelogenins are spherical 20 nm template proteins responsible for the nucleation of calcium phosphate to create dense layers of hydroxyapatite nanocrystallites [8]. Biomimetic remineralisation approaches focus on returning hydroxyapatite back into the enamel, together with amelogenin-based peptides to recover the hardness of the tooth. The nanohardness of enamel rods is reported to be 4 GPa [9], when present in bulk the enamel has a hardness comparable to that of window glass [10].

The field of ‘nanodentistry' has demonstrated emerging nanomaterials for periodontal and endodontic treatments. Healthy gums have a pocket depth of <4 mm between the gums and teeth [11], therefore enhanced penetration of nanoparticles into these surrounding dental tissues is anticipated. This is the same for root canal therapy, which should be disinfected up to the apical constriction, the narrowest opening of the canal with a diameter of 0.5–1.5 mm [12]. Nanoparticles such as metals or metal oxides could be intrinsically bactericidal or formulated to encapsulate drugs within polymers to enhance drug aqueous solubility for transportation into bacteria [13], and to achieve controlled release. The high surface area to volume ratio also allows for multiple drug loading that can result in synergistic antimicrobial efficacy, overcoming bacterial resistance [13].

Meanwhile, nanoparticles are increasingly used in resin matrix of prosthodontic and orthodontic composites, filling up gaps to increase the filler load, thereby reducing polymerisation shrinkage and increasing their mechanical properties [14]. Bond strength, flexural strength, compressive strength, fracture toughness and hardness are subsequently shown to be improved with the bulk use of mechanically strong nanoparticles, such as carbon nanotubes [15]. This current review presents an updated summary of non-exhaustive emerging nanomaterials for dental treatments in different areas, through rationalising the characteristics of each nanomaterial suited for its dental application. The challenges of nanomaterials used in dental treatments are also discussed, together with a future perspective on nanodentistry.

## Remineralisation treatment and preventive dentistry

Dental caries develops because of prolonged plaque accumulation whilst dental erosion is caused by dietary acids or gastro-oesophageal reflux disease. Hydroxyapatite (HA) nanoparticles are useful to counter the loss of enamel nanocrystals as they have similar morphology, crystallinity and chemical composition (Ca_10_(PO_4_)_6_(OH)_2_) [16]. HA nanoparticles can act as a filler to repair small depressions on the enamel, as they increase the surface area for binding [17], hence enabling stacking of the nanocrystallites. For example, it is reported that HA with a size of 20 nm suitably occupies space within the nanodefects caused by acidic erosion [18]. The deposited and adsorbed HA nanoparticles then are able to form a new biomimetic mineral coating [19–22].

Some lesions are non-cavitated, remaining relatively intact due to the superficial remineralisation by saliva. Preventive dentistry encourages the remineralisation of these subsurface lesions to preserve the tooth structure, function and aesthetics [23]. Nanoparticles can bypass the superficial layer, which acts as a diffusion barrier against subsurface uptake of minerals [24]. This pseudo-intact surface layer is reported to be permeable to ions too [16]. Nanoparticles serving as a calcium phosphate reservoir maintain supersaturation surrounding enamel minerals, thereby enhancing remineralisation [25–29]. HA substituted with magnesium, strontium and carbonate makes it more reactive for calcium ion release on the enamel [30], due to disruption in the crystalline lattice caused by elemental substitutions. On the other hand, amorphous calcium phosphate (ACP) offers better biodegradability than HA [31,32], due to its disordered structure and higher energy state.

ACP has been stabilised by casein phosphopeptides (CPP) derived from milk protein. Together they form CPP-ACP complexes which are reported to be 4 nm in diameter and have anti-cariogenic effects [33,34]. This is due to the ability of the phosphorylated amino acid cluster sequence [–Ser(P)–Ser(P)–Ser(P)–Glu–Glu–] within CPP to bind and stabilise calcium phosphate in the amorphous state, in addition to binding to dental plaque and enamel [35,36]. CPP-ACP is soluble in saliva, creating a concentration gradient that enables diffusion and localisation in supragingival plaque [29]. A cariogenic attack that gives rise to low pH conditions would facilitate the release of calcium and phosphate ions [29], to then be re-precipitated on the enamel surface.

There are studies that show the efficacy of HA over ACP in remineralisation [37,38], and vice versa [39]. To speed up the remineralising process, electrophoresis has been introduced to draw these particles into dental lesions [40–42]. These calcium phosphate nanoparticles formulated in toothpastes and mouthwashes not only treat dental hypersensitivity but help to achieve teeth-whitening [43]. Such cosmetic purpose has also been performed with titanium dioxide nanoparticles, which is an effective whitener [43]. Irradiated with blue light, polydopamine-modified titanium dioxide nanoparticles have achieved similar whitening effect compared with conventional whitening agents such as hydrogen peroxide, but with remarkably less damage on the enamel structure [44].

Meanwhile, plug-like deposition of nano-bioactive glass (calcium sodium phosphosilicate, 20–30 nm, spherical) within dentinal tubules has also been established to treat dentine hypersensitivity [45]. Glass ionomer cement, a dental restorative material containing aluminofluorosilicate glasses in a cross-linked matrix of polyacrylic acid as the ionomer bonds well chemically with dental hard tissues and enables fluoride release into lesions [46,47]. To enhance the mechanical properties further, it has been modified by reducing the size of the glass powder and incorporating the cement with nanosized HA and other nanoparticles such as zirconia [46,47].

To detect caries, nanoparticles made from fluorescein-labelled food-grade starch have been developed, which fluoresce when illuminated by a standard dental curing light and subsequently degrade into non-toxic compounds [48]. Efforts have also been made to encapsulate fluorescence dyes in calcium phosphate nanoparticles [49]. It is therefore anticipated that fluorophore-doped calcium phosphate nanoparticles will have the potential to detect early carious lesions and to directly impact dental treatment, giving these nanomaterials theranostic properties.

Preventing the formation of oral biofilm is also a part of preventive dentistry. In general, to penetrate an overall negatively charged matrix of extracellular polymeric substances into biofilm, the nanoparticles have to be positively charged [50] with particle sizes smaller than 130 nm [51]. Meanwhile, particle shape plays a role, as nano-blades on the edges of nanomaterials such as graphene oxide [51], and surface protrusion with nanotipped spines [52] can puncture bacterial cell membranes, inducing leakage of intracellular constituents and cell death.

**Figure 1.**
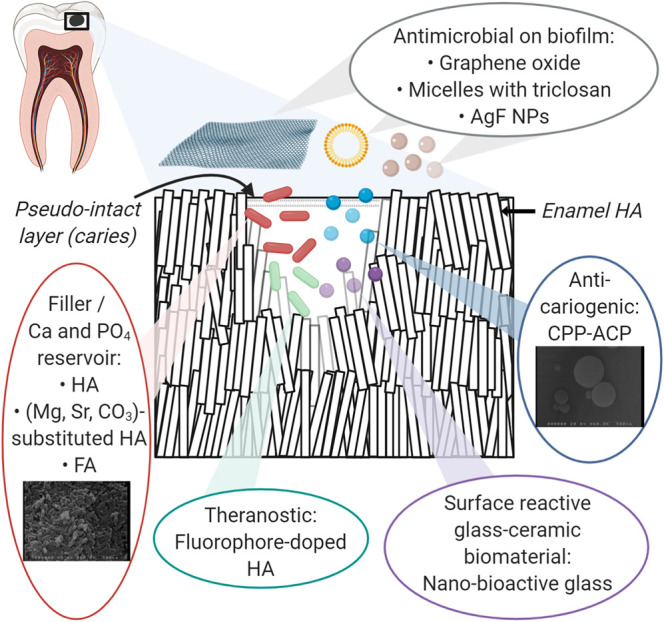
An overview of nanomaterials used in remineralisation treatment and preventive dentistry.

Hybrid nanomaterials for sustained release of antimicrobial drugs and an enhanced affinity towards enamel have been developed. For example, a polymeric-based micelle system (Pluronic® P123) contains triclosan as the antimicrobial mediator and diphosphoserine and pyrophosphate as tooth binding agents [53]. Another example is silver fluoride nanoparticles, whereby silver interacts more intensely with *Streptococcus mutans* due to its greater surface area [54]; and fluoride forms fluorapatite (FA), which has a lower critical pH than HA to resist dissolution. Sodium fluoride is also loaded in chitosan, which also has inhibitory effects on *S. mutans* [55], via ionic gelation of tripolyphosphate nanoparticles to deliver fluoride more effectively [56]. A hybrid nano-formulation containing silver fluoride and chitosan combining all the advantages above has also been presented [55]. A summary of nanomaterials used in remineralisation treatment and preventive dentistry is provided in Figure 1.

## Periodontal treatment

Periodontal disease refers to specific diseases that affect the gingiva, the supporting connective tissue and alveolar bone, which hold the teeth in the jaws [57]. Nanoparticles can be used to encapsulate drug molecules and enable delivery to localised areas affected by periodontal disease. This approach can reduce dosage-related side effects by selectively depositing the controlled amount of drug in the proximity of the area of interest [58]. A timely release of drugs by controlled disintegration is also useful. For example, Arestin® (minocycline microspheres) provides a long-term sustained release of minocycline to the periodontium. However, being microspheres, they may not penetrate deeper lesions in severe periodontitis [59].

Chitosan, a cationic naturally occurring polymer, is regarded as suitable for periodontal treatment. It has bioadhesive and antimicrobial properties which offer the palliative effects of an occlusive dressing and to deliver antiseptics such as chlorhexidine, metronidazole and nystatin [60]. A chitosan-based hydrogel containing triclosan, an antimicrobial drug prepared as nanoparticles using poly-ε-caprolactone, and flurbiprofen, an anti-inflammatory drug, gives rise to dual antibacterial and anti-inflammatory actions for localised treatment of periodontitis [61].

A combination of liposomes and ultrasound is also explored to deliver plasmid DNA into the gingiva, an endeavour to up-regulate neovascularisation and cell proliferation [62]. Liposome-encapsulated superoxide dismutase, an enzymatic inhibitory agent of neutrophil-mediated inflammation [63] can suppress periodontal inflammation in beagle dogs [64]. Oraqix®, a liposomal lidocaine/prilocaine has the potential to be used for non-invasive anaesthesia in place of local anaesthetic injections in periodontal therapy, which usually involves supra and/or subgingival scaling and root planning [65]. This could reduce pain and discomfort, subsequently reducing dental fear.

Ozone (O_3_) is a relatively safe antiseptic agent, as ozonated water will degrade back into oxygen without generating harmful residues; however, it has a half-life of only ∼20 min [66,67]. Ozone nano-bubble water has been developed, which remains stable for more than 6 months in storage in electrolyte solution [68]. Stability is provided by positive ions in the electrolyte solution concentrating around the gas nucleus due to its negatively charged surface (OH^−^ ions predominantly over H^+^ ions) and acting as a shell that prevents gas from dispersing [66].

Besides, area-specific configured nanorobots could also help in destroying bacteria in plaque [58]. Nanorobotic dentifrice, currently a hypothetical and theoretical microscopic device to be delivered by mouthwash or toothpaste, could patrol all supra and subgingival surfaces at least once a day, metabolising confined organic matter into non-toxic and odourless vapours and performing continuous calculus debridement [69].

**Figure 2.**
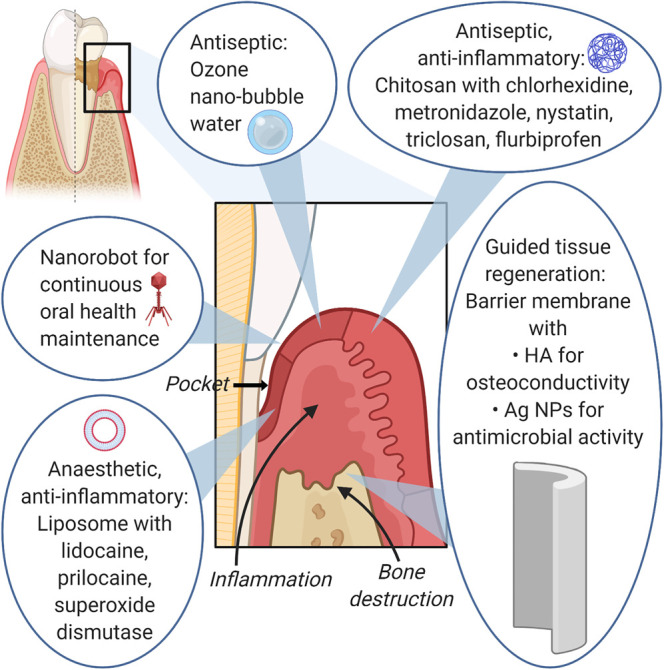
An overview of nanomaterials used in periodontal treatment.

Guided tissue regeneration used in the repair of periodontal defects employs a barrier membrane around the periodontal defect to deter epithelial downgrowth and fibroblast transgrowth into the wound site, so that there is space for true periodontal tissue regeneration [70]. HA nanoparticles, silver nanoparticles and nanodiamonds have been incorporated into these membranes to improve biocompatibility, osteoconductivity [71,72], antimicrobial properties [73] and mechanical properties of the membrane [74]. Figure 2 illustrates examples of nanomaterials used in periodontal treatment.

## Endodontic treatment

Endodontics involves the diagnosis and treatment of disease of the tooth pulp, which is the loose connective tissue in the centre of the tooth that forms and supports dentin [75]. Endodontic therapy, also known as root canal therapy, removes diseased and dead pulp tissues. However, the microorganisms causing primary infections tend to resist the intracanal antimicrobial procedure and secondary infections can be caused by microorganisms that are introduced during or after filling of root canals [75]. Nanoparticle-based disinfection with chitosan, zinc oxide and silver have been introduced in endodontics to provide more effective removal of microorganisms [76].

Chitosan, with its polycationic structure, binds to negatively charged bacterial cell walls due to the presence of carboxyl, phosphate and amino groups, altering membrane permeability and attaching to bacterial DNA and inhibiting replication [77]. Chitosan also chelates the trace metal elements that combine with cell wall molecules of microorganisms, destabilising the cell wall [78].

Zinc oxide nanoparticles under UV illumination generate reactive oxygen species (ROS) including hydrogen peroxide (H_2_O_2_), hydroxyl radicals (HO^•^) and superoxide (O^2−^) [79]. Superoxide and hydroxyl radicals, due to their negative charges, stay on the outer surface of the bacteria. Meanwhile, hydrogen peroxide molecules can pass through the cell wall to cause oxidative damage to cellular structures [79]. The uptake of toxic dissolved zinc ions also depletes intracellular ATP production and disrupts DNA replication [79].

Silver nanoparticles anchor to and infiltrate bacteria cell wall, then, electrostatic attraction between silver nanoparticles and sulfur-, nitrogen- or oxygen-containing functional groups on the cell membrane [80] causes physical membrane damage and cellular leakage [81]. Silver nanoparticles also produce high levels of ROS, together with dissolved silver ions they can increase cellular oxidative stress in microorganisms [81].

Root canal therapy has a success rate up to 86–98% [82]. Regenerative endodontic approaches have been trialled in order to instead regrow healthy pulp tissues. Rebuilding pulp tissues at the nanoscale level is important as it condenses multiple functionalities in a restricted volume and allows a better targeted delivery of bioactive molecules to the dental pulp [83]. For example, nano-assemblies of two polymers, poly-l-lysine dendrigraft (DGL) and poly-glutamic acid (PGA), with an anti-inflammatory hormone called α-melanocyte stimulating hormone (α-MSH) have been synthesised [83]. These multi-layered nano-assemblies reduce the inflammation of pulp connective tissues and promote their regeneration by promoting adhesion and multiplication of pulp fibroblasts [84].

**Figure 3.**
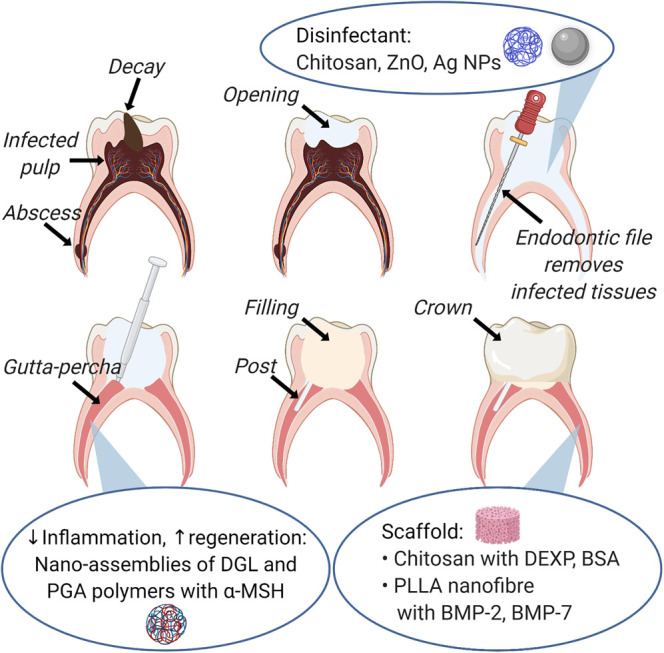
An overview of nanomaterials used in endodontic treatment.

Regenerative endodontic therapy can be enhanced by scaffolds to which stem cells from the apical papilla (SCAP) can attach, proliferate and differentiate [85]. For this purpose, the increased surface area of nanoparticles is useful for cell adhesion and biological activity. They can also be developed into a controlled-release system with growth factors to support and regulate the differentiation of stem cells [83]. For example, mechanically strong chitosan nanoparticles have been shown to improve SCAP adherence, viability and differentiation [86]. Controlling the alignment and orientation of chitosan nanofiber from electrospinning will also improve the material strength [87]. A nanofiber scaffold system of chitosan nanoparticles loaded with dexamethasone sodium phosphate (DEXP) reduces inflammation [88], and when loaded with bovine serum albumin (BSA) maintains the osmotic pressure and transportation of nutrients into cells for bone tissue regeneration [89,90]. Similarly, an injectable scaffold of poly-l-lactic acid (PLLA) nanofibrous microspheres with controlled release of bone morphogenic protein 2 (BMP-2) helps in the promotion of SCAP differentiation into odontoblast-like cells [91]. These nanomaterials used in endodontic treatment are presented in Figure 3.

## Prosthodontic treatment

Prosthodontics is the branch of dentistry that deals with the functional and aesthetic restoration and replacement of teeth and maxillofacial tissues. It involves complete dentures, fixed and removable partial dentures, maxillofacial prosthetics and implants [92]. A denture base material of choice, with over 95% of use in complete dentures, is poly(methyl methacrylate) (PMMA) [93]. It is heat-cured to form acrylic resin, is cheap, biocompatible with good physicochemical properties and acceptable aesthetics [93]. However, its surface porosity makes it prone to plaque accumulation [94], polymer fatigue failure and oral mucosa irritation [95].

The impregnation of metal oxide nanoparticles, such as titanium dioxide or iron (III) oxide has been shown to reduce the porosities of PMMA and hence reduce bacterial attachment [96]. Meanwhile, embedded titanium dioxide and silver nanoparticles have reduced the adherence of *Candida* species on denture resins [97,98]. Improvement in mechanical properties, such as reduced polymerisation shrinkage with carbon nanotubes [15], increased flexural strength with zirconium dioxide nanoparticles [99] and hardness with alumina nanoparticles [100] has been observed. Silica nanoparticles treated with 3-methacryloxypropyltrimethoxysilane (MPTS) have led to higher bond strength and better adhesion [15], with MPTS aiding the chemical bonding of silica nanoparticle filler to the resin during curing [94].

Titanium alloy and chrome cobalt are used in removable partial denture connectors, for skeletal protheses and fixed bases for crowns and bridges [101]. However, they are reported to have poor corrosion resistance, affect tooth mobility and may cause gingival inflammation [101,102]. Instead of polished titanium, titanium and zirconia nanoparticles have significantly reduced the number of adherent bacteria [103]. Meanwhile, cobalt and cobalt oxide nanoparticles have been recommended for their bactericidal properties [104,105].

Ceramics such as zirconia or alumina used to produce crowns and bridges fulfil aesthetic and functional requirements [106], but they are of low ductility and high brittleness [95]. Ormocer, short for ‘organically modified ceramics', have a matrix of ceramic polysiloxane with lower shrinkage compared with the PMMA matrix [107]. To further reduce polymerisation shrinkage to prevent secondary caries, silicon oxide nanoparticles are added as the chemical base for both the filler and resin matrices, which also increase the hardness of the restorative material [108]. Other nanofillers that have been trialled in composite resins include nanoparticles of alumina, zirconia, titania and carbon nanotubes [109].

Dental implants made of titanium are most often used, followed by zirconia implants. To improve implant–bone interconnection quality, including both mechanical anchorage and bone remodelling, nanotopographies can help by increasing surface wetness and stimulating continuous protein adsorption and the formation of blood components at implant interface [110]. This includes titania nanosheet structures fabricated on titanium surfaces [111], coatings of HA and alumina nanoparticles for good osteointegration [112], and nanocoating with quercitrin, a natural flavonoid, which reduces osteoclast activity [113]. Other nanoparticles, for example silver, zinc oxide, copper (II) oxide and chlorhexidine nanoparticles have also been used in dental implants for their antimicrobial properties [112].

**Figure 4.**
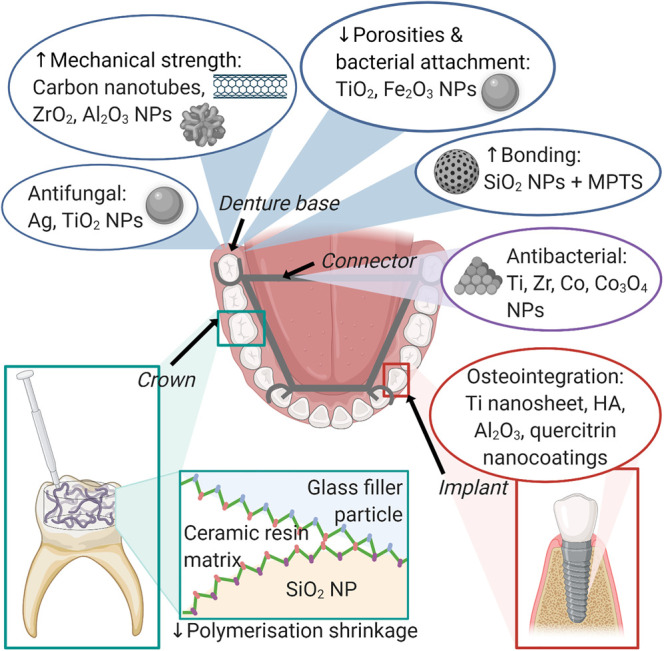
An overview of nanomaterials used in prosthodontic treatment.

Furthermore, patients with facial prostheses made from silicone experience *Candida albicans* infection [114]. Addition of silver nanoparticles increases the antifungal efficiency [114]. Titanium dioxide and silicon oxide nanoparticles also increase the mechanical properties of maxillofacial silicone materials [115,116]. A summary of nanomaterials used in prosthodontic treatment is presented in Figure 4.

## Orthodontic treatment

Orthodontics involves all preventive and corrective procedures of dental irregularities that requires the repositioning of the teeth by functional and mechanical means to establish normal occlusion and pleasing facial contours [117]. The use of braces interferes with tooth leading to the common side effect of white spot lesion formation [118]. Silver and titanium dioxide nanoparticles have been added to orthodontic composites in cementing brackets providing antibacterial effects [119]. Nanoparticles of HA and FA and fluoride-releasing elastomers have also been incorporated to counter enamel demineralisation adjacent to the brackets [119].

**Figure 5.**
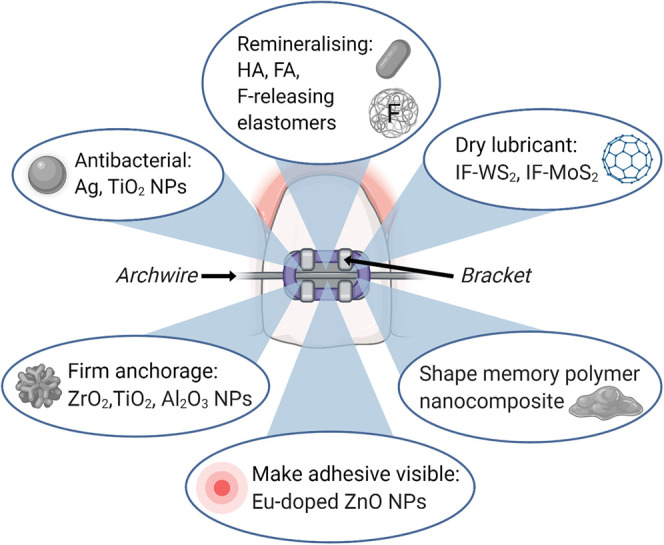
An overview of nanomaterials used in orthodontic treatment.

To reduce friction between bracket slot and archwire for efficient tooth movement, inorganic fullerene-like tungsten disulfide (IF-WS_2_) and molybdenum disulfide (IF-MoS_2_) nanoparticles have been coated on orthodontic wires as a dry lubricant [120,121]. For a firmer anchorage and increased mechanical strength, a combination of zirconia and titanium dioxide nanoparticles has been added to orthodontic adhesive [122] and alumina nanoparticles have been added to clear plastic polymer braces [123]. Making orthodontic adhesive visible with europium-doped zinc oxide nanoparticles increases safety by allowing complete removal of the adhesive after treatment [124]. Shape memory polymer, being responsive to body temperature or light by photoactive nanoparticles, can form a temporary shape with desired geometry and surface characteristics which can influence tooth movement [119,125]. These nanomaterials used in orthodontic treatment are summarised in Figure 5.

## Challenges faced by emerging dental nanomaterials

Most nanomaterials used for cleaning and remineralising would be delivered as suspension within mouthwash and dentifrice, whereas powdered nanoparticles would be present in dental composites. For the purposes of authorisation and monitoring of emerging nanomaterials, it is suggested that silver nanoparticles in a composite would likely fall under the category of a medicinal product, whereas HA in a denture would be regarded as a medical device [126]. The benefits of these emerging nanomaterials must be weighed against the risks, especially to host cells and homeostasis of the oral cavity of the patients. For example, although designed to be inert in the oral environment, rapid leaching of silver nanoparticles that are directly incorporated into resin-based composite has been reported [127], and ROS generation from these accumulated nanoparticles would lead to increased pro-inflammatory reactions and oxidative stress [128].

Overloaded nanoparticles trapped in mucous secretion of the saliva may trigger a local hypersensitivity reaction in the oral epithelium and interact with salivary components. For example, silica nanoparticles have been shown to induce conformational changes of lysozyme and amylase and compromise their enzymatic functions [129]. Accidental ingestion of these nanomaterials, with the chance of increased rate of absorption, requires further investigation. For example, small amounts of ingested titanium dioxide nanoparticles might be absorbed from the gastrointestinal tract into systemic circulation, potentially affecting vital organs in the body [130]. Apart from the risks to patients, occupational exposure to dental practitioners, such as inhalation of aerosols from drilling into a nanocomposite, has also been highlighted [126].

## Conclusion

Application of nanotechnology in all areas of dentistry is emerging, with benefits arising from small particle size for enhanced permeation into deeper lesions, large surface area to volume ratio for enhanced bioactivity such as osteointegration, controlled release of bioactive molecules reducing dosage and resulting in lesser side effects, and site-targeted delivery of growth factors for localised regenerative treatment. Whilst research in this review focuses on the benefits of nanomaterials intended for use in the oral cavity, the general risks of nanomaterials in all healthcare areas remain a concern and will require specific and long-term investigation of safety. The application of nanotechnology in dentistry is anticipated to grow further, and as such, an interdisciplinary approach encompassing expertise in nanotechnology-based material science and dentistry is required.

## Summary

- The nanostructure of the tooth surface and the inherent properties of nanoparticles initiate the emergence of nanomaterials in dentistry.
- Various forms of calcium phosphate nanoparticles and nano-bioactive glass have been explored to return minerals into the teeth for remineralisation and caries prevention.
- Nanoparticle formulations encapsulating antiseptic, anaesthetic, anti-inflammatory and osteointegration-promoting agents have been developed for site-targeted and controlled delivery.
- Nanoparticles in dental composites help to improve their bonding and reduce friction, lower porosity and polymerisation shrinkage and improve their mechanical strength.
- More nanomaterials for dental treatments will emerge in the foreseeable future for added benefits to conventional dental materials.

## Open access

Open access for this article was enabled by the participation of King's College London in an all-inclusive *Read & Publish* pilot with Portland Press and the Biochemical Society under a transformative agreement with JISC.

## Abbreviations

ACP: amorphous calcium phosphate
CPP: casein phosphopeptides
FA: fluorapatite
HA: hydroxyapatite
MPTS: 3-methacryloxypropyltrimethoxysilane
PMMA: poly(methyl methacrylate)
ROS: reactive oxygen species
SCAP: stem cells from the apical papilla
